# Genome-wide identification of the pectin methylesterase inhibitor genes in *Brassica napus* and expression analysis of selected members

**DOI:** 10.3389/fpls.2022.940284

**Published:** 2022-07-22

**Authors:** Duoduo Wang, Shunda Jin, Zhe Chen, Yue Shan, Lei Li

**Affiliations:** ^1^College of Chemistry and Life Sciences, Zhejiang Normal University, Jinhua, China; ^2^School of Integrative Plant Science, Cornell University, Ithaca, NY, United States; ^3^Key Laboratory of Tropical Fruit Tree Biology of Hainan Province, Haikou, China; ^4^School of Life Sciences, Jiangsu University, Zhenjiang, China

**Keywords:** *Brassica napus*, genome-wide, pectin methylesterase inhibitor, evolution, plant cell wall, expression patterns, Sclerotinia sclerotiorum

## Abstract

Pectin methylesterase inhibitors (PMEIs) modulate the status of pectin methylesterification by inhibiting the activity of pectin methylesterase (PME). Recent advances indicate PMEIs play an important role in regulating plant cell wall properties and defense responses. In this study, a genome-wide analysis of *PMEI* gene family in *Brassica napus* (*B. napus*) was conducted and the expression patterns of *PMEI* genes in response to *Sclerotinia sclerotiorum* (*S. sclerotiorum*) was investigated. A total of 190 PMEI proteins were identified from the genome of *B. napus*. Chromosomal location, gene structure and properties of the PMEI family were analyzed, and these features were compared with *Arabidopsis thaliana (A. thaliana)*. A total of 123 syntenic ortholog pairs were detected from *BnPMEI* family by synteny analysis. Results showed the expansion of *BnPMEI* genes was likely predominately from whole-genome duplication (WGD) or segmental duplications. Multiple *cis*-elements related to plant growth and development, environmental stress responses, hormone responses were detected in the promoters of *BnPMEI* genes, implying they were regulated by both internal and external factors. Furthermore, expression analysis of transcriptome data combined with quantitative RT-PCR (qRT-PCR) validation identified several candidates that were strongly responsive to *S. sclerotiorum* infection. These *BnPMEI* genes are candidates for manipulation to breed novel and improved genotypes that are more resistant to sclerotinia stem rot (SSR). Extensive interactions were detected among 30 BnPMEI proteins, forming complex protein-protein interaction networks. Besides, 48 BnPMEIs showed interactions with other proteins including a range of cell wall structure-related enzymes. This study provides new insights into the evolution and function of PMEIs in *B. napus* and lays a foundation for breeding novel genotypes for crop improvement.

## Introduction

The plant cell wall is a complex network composed of polysaccharides, including cellulose, hemicelluloses and pectin, as well as other structural proteins. Cell walls are organized into a three-dimensional matrix and play an important role in biological processes including development and disease resistance (Höfte and Voxeur, 2017; Jamet and Dunand, 2020). Pectin, the most abundant and structurally complex polysaccharide, is generally classified into three major types: homogalacturonan (HG), rhamnogalacturonan I (RG-I), and rhamnogalacturonan II (RG-II) (Mohnen, 2008). HG is highly methylesterified when secreted to cell wall matrix, and methylesters can be removed from HG by pectin methylesterases (PMEs) (Levesque-Tremblay et al., 2015). Pectin methylesterases inhibitors (PMEIs), belonging to large multigene family in plant species, inhibit the activity of PMEs by forming a reversible 1:1 complex (Giovane et al., 2004; Jolie et al., 2010). The pattern and degree of pectin methylesterification are tightly regulated by localized expression of specific PMEs and PMEIs isoforms (Coculo and Lionetti, 2022). PMEI was first discovered in kiwi fruit (Balestrieri et al., 1990) and then detected in many other plant species including members of the Brassica family such as *Arabidopsis thaliana (A. thaliana)* (Raiola et al., 2004; Wormit and Usadel, 2018).

In recent years, the plant primary cell wall model has been revised, where pectin metabolism has been proposed to play a more crucial role in influencing cell wall traits than previously thought (Park and Cosgrove, 2012; De Lorenzo et al., 2019). Pectin methylesterification status impacts the biomechanical properties of cell wall and undergoes dynamic changes during plant development and in response to various environmental stresses (Wormit and Usadel, 2018). A strong link between PMEIs and plant development and defense responses was observed in many plant species including *A. thaliana* (Lionetti et al., 2017), rice (Nguyen et al., 2017), maize (Woriedh et al., 2013), and pepper (An et al., 2008). Genome-wide identification of *PMEI* gene family has been performed in a range of plant species including dicots including Arabidopsis (Wang et al., 2013), tomato (Jeong et al., 2018), *Brassica campestris* (Liu et al., 2018a), *Brassica rapa* (Tan et al., 2018), as well as monocot plants including rice (Nguyen et al., 2016), sorghum (Ren et al., 2019), and maize (Zhang et al., 2019).

*Brassica napus L (B. napus)*, a major oil crop in the world, is susceptible to various biotic stresses including sclerotinia stem rot (SSR) a devastating disease caused by *Sclerotinia sclerotiorum* a necrotrophic fungal pathogen. It seems likely that cell wall changes involving pectin metabolism could be involved in *S. sclerotiorum* infections and *PMEI* genes may play a role in stress-induced defense response in *B. napus*, but little information is available in the literature. Very recently, lower degree of pectin methylesterification was accompanied by lower expression level of *PMEI* genes in the leaves of cadmium (Cd) -tolerant rapeseed compared to Cd-sensitive genotype (Wu et al., 2021). This suggests PMEIs might regulate cadmium-induced stress response in *B. napus* through facilitating Cd retention in the cell walls.

In this study, we conducted a genome-wide identification of *BnPMEI* genes in *B. napus*. A total of 190 *PMEI* gene members were identified. Systematic analysis of the *BnPMEI* gene family included investigating phylogenetic relationships, gene structure, conserved motif patterns, gene duplication and cis-elements. Expression analysis of *BnPMEI* genes in response to *S. sclerotiorum* infection revealed several candidate genes including *BnPMEI19*, *BnPMEI76*, and *BnPMEI127* that were likely to regulate SSR-triggered defense. The work provides key information for future function characterization of BnPMEIs and serves as a basis for breeding novel genotypes with enhanced stress tolerance.

## Materials and methods

### Identification of *PMEI* genes in *Brassica napus*

*Brassica napus* genome sequences were downloaded from EnsemblPlants^1^. Firstly, BLASTP search was conducted in the Genoscope database^2^ (Chalhoub et al., 2014) to find the putative BnPMEI members using the 79 *A. thaliana* PMEI protein sequences as queries downloaded from TAIR^3^. Secondly, hmmersearch in the HMMER web server^4^ was performed to screen candidate *PMEI* gene sequences using the Hidden Markov Model (HMM) profile (PF04043) from the Pfam database^5^. To check the presence of the conserved PMEI domain in each protein, sequences of the putative PMEI proteins were validated through the Simple Modular Architecture Research Tool (SMART) database^6^ (Letunic et al., 2012), the NCBI Conserved Domain Database (Marchler-Bauer et al., 2011) and the Pfam database (Finn et al., 2016). The *PMEI* genes identified in the genome of *B. napus* were named according to their locations and orders on the chromosomes or scaffolds (Supplementary Table 1). Physicochemical properties including the length of protein sequence, molecular weight (MW), and isoelectric point (pI) were predicted using ExPASy website^7^. SignalP 4.1 Server^8^ was used to predict the signal peptide sequences. Trans-membrane hidden Markov model (TMHMM) Server V2.0^9^ was used to explore the transmembrane helices (Krogh et al., 2001). WoLF PSORT^10^ and ProtComp 9.0^11^ were used to predict the subcellular localization of BnPMEI proteins (Horton et al., 2007; Jing et al., 2017).

### Analysis of conserved motif and gene structure of the BnPMEI proteins

Conserved motifs of the *PMEI* gene family in *B. napus* were analyzed *via* the program MEME (Multiple Em for Motif Elicitation)^12^ using full length protein sequence of each PMEI member, with default parameters except for parameters: maximum number of motifs set as 5 and motif width set as 6–100 amino acid (Bailey et al., 2009). The exon-intron structures of the *PMEI* genes were illustrated with the online tool GSDS (Gene Structure Display Server)^13^ (Hu et al., 2015).

### Chromosomal locations and phylogenetic analysis

The chromosomal positions of the *BnPMEI* genes were retrieved from the Genoscope database, and were visualized using the TBtools software (Chen et al., 2020). Multiple sequence alignments of PMEI amino acid sequences were performed using the Muscle algorithm in MEGA11 software. Based on alignment results, phylogenetic trees were constructed using MEGA 11 by the maximum likelihood (ML) method with the following parameters: Jones–Taylor–Thornton (JTT) model, partial deletion, site coverage cutoff was 50%, and bootstrap replications set as 1000 to define the reliability of the resulting tree. The trees were visualized *via* the online tool Interaction Tree of Life (iTOL^14^).

### Gene duplication, synteny, and evolutionary analysis

The length of each chromosome and the location of each *BnPMEI* and *AtPMEI* gene were retrieved from the Genoscope and TAIR database. Multiple collinear scanning toolkits (MCScanX) were used to analyze gene replication events and synteny relationships in *B. napus* or between *B. napus* and *A. thaliana* (Wang et al., 2012). The collinearity of the paralogous gene pairs of *PMEIs* in *B. napus* was depicted using the Advanced Circos in TBTools. To illustrate the orthologous relationship of *PMEIs* between *B. napus* and *A. thaliana*, the syntenic map was constructed with the Dual Systeny Plotter in TBtools. The synonymous rate (*K*s), non-synonymous rate (*K*a), and *K*a/*K*s ratios of each gene pair were calculated using the Simple *K*a/*K*s Calculator in TBTools. The divergence time of homologous gene pairs was calculated by the following equation T = Ks/2λ (λ denotes the estimated clock-like rate of synonymous substitution that is 1.5 × 10^–^^8^ substitutions/synonymous site/year in dicots) (Blanc and Wolfe, 2004).

### *Cis*-elements prediction

To investigate the cis regulatory elements in the promoter regions of *BnPMEI* genes. The 2000-bp genomic DNA sequence upstream the translation start codon of each gene was extracted using TBtools, and cis-acting elements were predicted using the online tool PlantCARE^15^ (Lescot et al., 2002).

### Plant material and pathogen inoculation

The Resistant (R)-line ‘Zhen12F28’ and Susceptible (S)-line ‘Zhen11C11’ used in this work were kindly provided by Zhenjiang Academy of Agricultural Sciences. All plants were grown in a growth room under a photoperiod of 16 h of light and 8 h of dark at 22°C and 60–80% relative humidity. Four-leaf-stage rapeseed seedlings with similar growth rate were selected for inoculation. *S. sclerotiorum* isolate was washed with sterilized water and cultured on potato dextrose agar medium. Adaxial surface of the fourth-leaf from each seedling was inoculated with 5-mm diameter mycelial agar plugs punched from the growing margin of a 3-day-old culture of *S. sclerotiorum*. Mock-inoculated plants were treated with 5-mm diameter agar plugs only. Plants were incubated in a sealed and humidified chamber for developing disease symptoms. Leaf tissue around the inoculation site was collected at three time points (24, 48, and 96 h post inoculation). Three biological replicates were sampled for each treatment.

### Phenotyping, trypan blue and 3,3’-diaminobenzidine staining

Images of inoculated leaves were taken using a camera and lesion area was calculated using Image J. Cell viability was tested by Trypan blue staining and hydrogen peroxide (H_2_O_2_) *in situ* was detected by DAB (0.5 mg/ml) staining as described (Wang et al., 2009, 2014). Images were taken using a Leica DM IL LED (LEICA, Germany) invert microscope under bright-field.

### Expression analysis of *BnPMEIs* using RNA-seq data and quantitative RT-PCR

Transcriptome data of *B. napus* under *S. sclerotiorum* stress was obtained from NCBI SRA database under the following projects (ID: PRJNA321917; PRJNA274853). Fold change was expressed as ratio of Fragments Per Kilobase per Million (FPKM) values in the treated group to control group, and heatmap was generated based on log 2 of fold change value using TBtools software. Tissue-specific expression profiles of the *BnPMEIs* genes was performed. Expression profiles of the 190 *BnPMEI* genes were compared between five different tissues of *B. napus* including root, stem, young leaves, petals and silique pericarp at full-bloom stage using public data obtained from Brassica EDB^16^.

Quantitative RT-PCR (qRT-PCR) assays were performed to investigate the expression levels of ten selected candidates in the R-line and S line at different time points post inoculation, as well as in different tissues including root, stem, leaf, petal and silique at full-bloom stage. The 10 genes include *BnPMEI168, BnPMEI145, BnPMEI19, BnPMEI41, BnPMEI161, BnPMEI46, BnPMEI76, BnPMEI128, BnPMEI127*, and *BnPMEI64* (Supplementary Table 2).

For the qRT-PCR experiments, total RNA was extracted using PureLink Plant RNA Reagent (Invitrogen, Carlsbad, CA, United States) kit. First cDNA synthesis was performed using MonScript™ RTIII All-in-One Mix with dsDNase (Monad). qPCR was performed using ChamQ Universal SYBR qPCR Master Mix (Vazyme Biotech Co., Ltd) according to users’ guide. The qPCR cycling was set to 95°C for 3 min, 40 cycles of (95°C for 15 s, 60°C for 30 s, 72°C for 15 s) using a LightCycler480 II instrument (Roche, Ltd). Actin from *B. napus* was used as internal reference. Primers used for QPCR were list in Supplementary Table 2. Relative gene expression was calculated according to the 2^–ΔΔ^*^Ct^* method (Livak and Schmittgen, 2001). Each treatment involved three biologically independent RNA samples and each qPCR assay was performed with three technical replicates.

### Analysis of uronic acid contents and cell wall-bound methyl ester contents

Acetone insoluble solids (AIS) were prepared with *B. napus* root, stem, leaf, petal and silique at full-bloom stage. Tissues were homogenized with 80% of cold acetone using a Polytron Homogenizer (VWR). The insoluble residues were collected by filtering the mixture through Miracloth (VWR), followed by washed successively with 100% acetone to remove all pigment. The powder was left overnight to dry at room temperature (RT). A total amount of 100 mg AIS was used for preparing pectin enriched fractions (PEFs). Briefly, the AIS was treated with 50 mM ammonium oxalate solution for 24 h at RT, and soluble fractions were collected by centrifugation and lyophilization. The lyophilized samples were treated with 4N potassium hydroxide for 24 h at RT, and were then centrifuged and lyophilized to obtain PEFs. Uronic acid content in the PEFs was measured as described in Jeong et al. (2018). Cell wall-bound methyl ester contents were assayed by measuring the methanol released from the cell wall after saponification treatment. Methanol reacted with 2,4-Pentanedione to form product with absorbance at 412 nm. Absorbance was measured using a spectrophotometer, and methanol content was determined by interpolating from a standard curve generated with a dilution series of methanol. Degree of pectin methyl esterification of cell walls was expressed as ratio of value of cell wall-bound methanol to value of uronic acid content.

### Gene ontology analysis and protein–protein interaction prediction

Each BnPMEI protein was analyzed using InterproScan 5^17^ for GO category annotation (Hunter et al., 2009). Functional interaction networks of BnPMEIs were generated using the STRING database which integrates all public sources of proten–protein interactions (PPI) and computational predictions and predicts direct (physical) and indirect (functional) associations. Proteins interacting with each of the BnPMEI member were predicted using the STRING database by submitting each protein sequence independently (Szklarczyk et al., 2011).

### Statistical analysis

The statistical analysis was performed with ANOVA using Graphpad Prism 5. Error bar was represented as mean ± standard error of mean (SEM). Each comparison was performed at a significance level *P* = 0.05.

## Results

### Identification and physicochemical properties of the *PMEI* gene family in *Brassica napus*

Using BLAST, 190 PMEIs were acquired from the reference genome of *B. napus* by BLASTP searches with 79 AtPMEI sequences from *A. thaliana* as queries. In addition, the genome annotation data of *B. napus* was searched against the global Hidden Markov Model (HMM) profile of the conserved PMEI domain (Pfam04043) using HMMER 3.0 web server. Combining the two methods, a total of 190 sequences were obtained as members of the *PMEI* gene family. The candidates were verified to cover the conserved domain of PMEI using SMART, CDD and Pfam. The *BnPMEI* genes were then renamed based on their locations and orders on the chromosomes. Detailed information of gene name, ID, chromosomal locations, protein sequence length, MW, pI and their homologs in *A. thaliana* were listed in Supplementary Table 1. The length of the 190 PMEI proteins ranged from 65 to 356 amino acids (AA), with an average length of 189 AA. The MW ranged from 6901.94 to 38796.47 Da with an average of 20757.24 Da. The pI ranged from 4.18 to 11.87. Signal peptide sequence and transmembrane helices of each BnPMEI were predicated using SignalP 4.1 Server and TMHMM Server V2.0, respectively. Signal peptide sequence was present in 177 BnPMEIs. A total of 70 BnPMEI were expected to possess one transmembrane helix, while only BnPMEI37 had two transmembrane helices (Supplementary Table 3). This suggested BnPMEIs with at least one transmembrane helix were anchored on cell membrane. The remaining BnPMEI proteins were completely exported to the extracellular matrix. Subcellular localization prediction using WoLF PSORT and ProtComp 9.0 revealed the majority of BnPMEIs were located in the extracellular cell wall matrix (Supplementary Table 3). In *A. thaliana*, the AtPMEI proteins were also predicted to be secreted to cell wall (Wang et al., 2013), which was consistent with the predicted locations of BnPMEIs.

### Phylogenetic analysis

**FIGURE 1:**
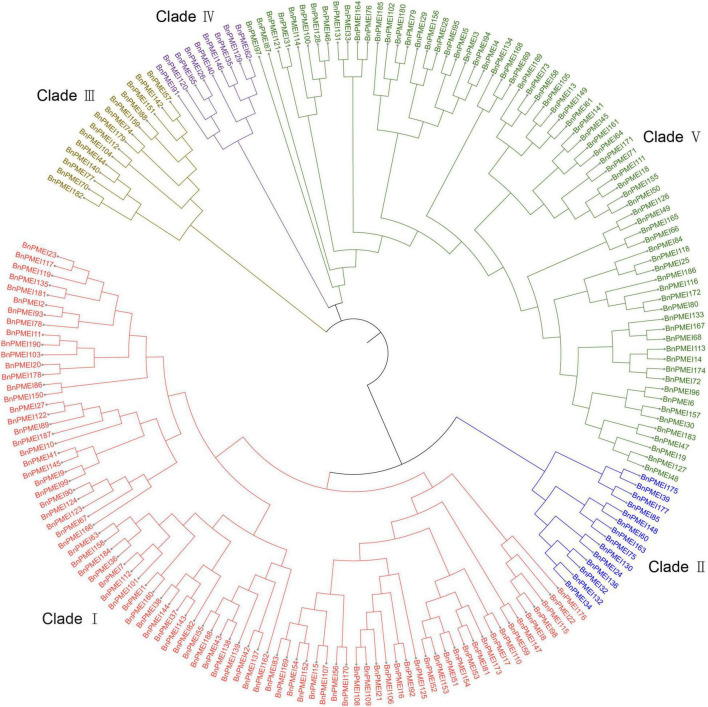
Phylogenetic tree of PMEI proteins in *B. napus*. The maximum likelihood (ML) tree was constructed using MEGA11 with full-length amino acid sequences of 190 BnPMEIs, and the bootstrap replicate was set as 1000 times. Different clades of PMEI family were represented by different colors.

**FIGURE 2:**
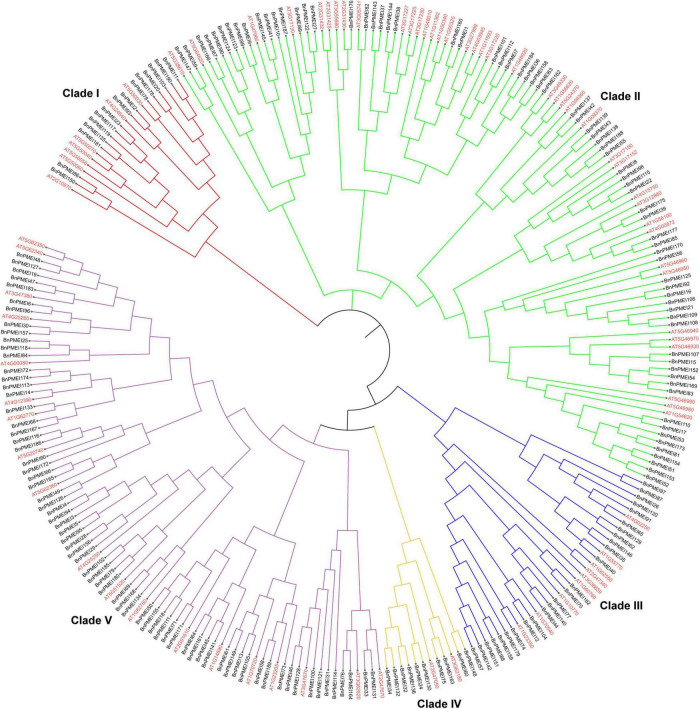
Phylogenetic analysis of PMEI proteins in *B. napus* and *A. thaliana*. The unrooted maximum likelihood (ML) phylogenetic tree was constructed using full-length amino acid sequences of 267 PMEI proteins from both species with MEGA11. Gene IDs in red color represented *PMEIs* from *A. thaliana*, and gene names in black indicated *PMEIs* from *B. napus*. The arcs in different colors indicated different clades of the PMEI proteins.

To understand the evolutionary relationships among the PMEIs in *B. napus*, a phylogenetic tree was constructed based on the alignment of BnPMEI protein sequences. The BnPMEIs were classified into five clades. Clade I was the largest clade with 82 members, followed by clade V which was composed of 71 members. The remaining three clades (Clade II, Clade III, and Clade IV) contained 14, 14 and 9 BnPMEIs, respectively (Figure 1). In order to analyze the relationship between PMEIs from *B. napus* and *A. thaliana*, an unrooted tree was constructed using the full-length amino acids of 267 PMEIs from both species (Figure 2). Here, the 267 PMEIs were clustered into five clades, which was consistent with the classification of BnPMEIs. Among these PMEIs, clade II contained the largest number of PMEIs (111). Besides, 22 members belonged to clade I, 34 to clade III, 12 to clade IV and 88 to clade V. Each clade contained both BnPMEIs and AtPMEIs.

### Characterization of gene structure and conserved motifs of BnPMEI proteins

Gene structure plays an important role in gene function divergence (Xu et al., 2012). In this study, the exon-intron structure of the 190 *BnPMEI* genes was analyzed using the web server GSDS (Gene Structure Display Server)^18^. Results revealed most of the *BnPMEI* genes (140/190) contained only one exon in their DNA sequences without any intron disrupting the coding sequence (Supplementary Figure 1). The number of exons ranged from one to six with an average of 1.3 exon. *PMEI* genes in Arabidopsis contained 1.3 exons on average as well, which was in line with this study (Wang et al., 2013). Forty-seven genes were found with two exons. Three *BnPMEI* genes contained more than two exons, including *BnPMEI190* which had 3 exons, *BnPMEI166* containing 4 exons, and *BnPMEI74* with 6 exons, respectively. Conserved motifs frequently present in the BnPMEI proteins were constructed using the MEME (Multiple Em for Motif Elicitation) (Supplementary Figure 1). The sequence and length information of the top five motifs were shown in Supplementary Figure 2. Motif 2 was found in BnPMEIs from all of the five clades, whereas motif 4 was only detected in BnPMEIs from Clade V. PMEIs belonging to Clade I, Clade IV, and Clade V contained motif 1, 2, 3, 5. PMEI proteins from Clade III had motif 1, 2, 3. Motif 2 and 5 were detected in BnPMEI members from Clade II. Interestingly, BnPMEIs within the same clade had similar motif composition and exon-intron structure, strongly supporting the reliability of clade classification by the phylogenetic analysis.

### Chromosomal distribution, genome synteny and gene duplication

**FIGURE 3:**
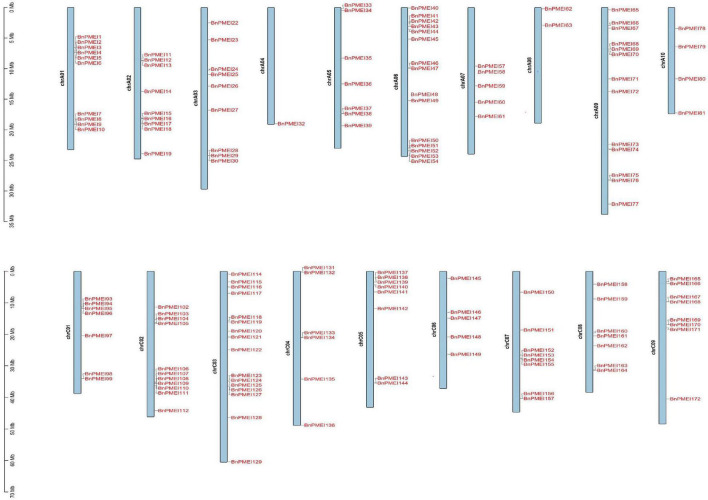
The distribution of *BnPMEI* genes on *B. napus* chromosomes. A total of 151 *BnPMEI* genes were mapped to the 19 chromosomes, and the remaining *BnPMEI* genes were unassembled scaffolds. The chromosome number was indicated on the left side of each chromosome and gene names were shown on the right side of each chromosome. The scale was megabases (Mb). Chr, chromosome.

*Brassica napus* (AACC, 2n = 38) is originated in the Mediterranean region about 7500 years ago by natural hybridization between two diploid progenitors, *Brassica rapa* (AA, 2n = 20) and *Brassica oleracea* (CC, 2n = 18). *B. napus* has 19 chromosomes, of which 10 chromosomes are from An subgenome and 9 from Cn subgenome (Chalhoub et al., 2014). The smallest chromosome is A10 while the largest chromosome is C03. Both A and C subgenomes of *B. napus* have undergone duplications as reported (Chalhoub et al., 2014). Chromosomal location analysis showed the *BnPMEI* genes were unevenly distributed across 19 chromosomes. Locations of 151 *BnPMEI* genes were confirmed on the 19 chromosomes (Figure 3). Owing to the incomplete information of *B. napus* genome, 29 *BnPMEI* genes were assigned to random chromosomes (11 on Ann random chromosomes, 17 on Cnn random chromosomes and 1 on Unn random chromosome). In terms of the remaining *BnPMEI* genes, the chromosomes they were located on were already known but the exact locations they resided were unknown. In the *BnPMEI* gene family, 92 and 98 *BnPMEI* genes were located on A and C subgenomes, respectively. The number of *BnPMEI* genes per chromosome was from 1 to 16. The maximum number of *BnPMEIs* was discovered on chromosome C03 while the minimum number was on chromosome A04 with only one. Chromosome A01, A06, A09, C02, and C03 contained more than 10 *BnPMEI* genes. The number of *BnPMEIs* was not positively correlated with chromosome length. Also, clusters of *BnPMEI* genes were detected on diverse chromosomes (Figure 3).

**FIGURE 4:**
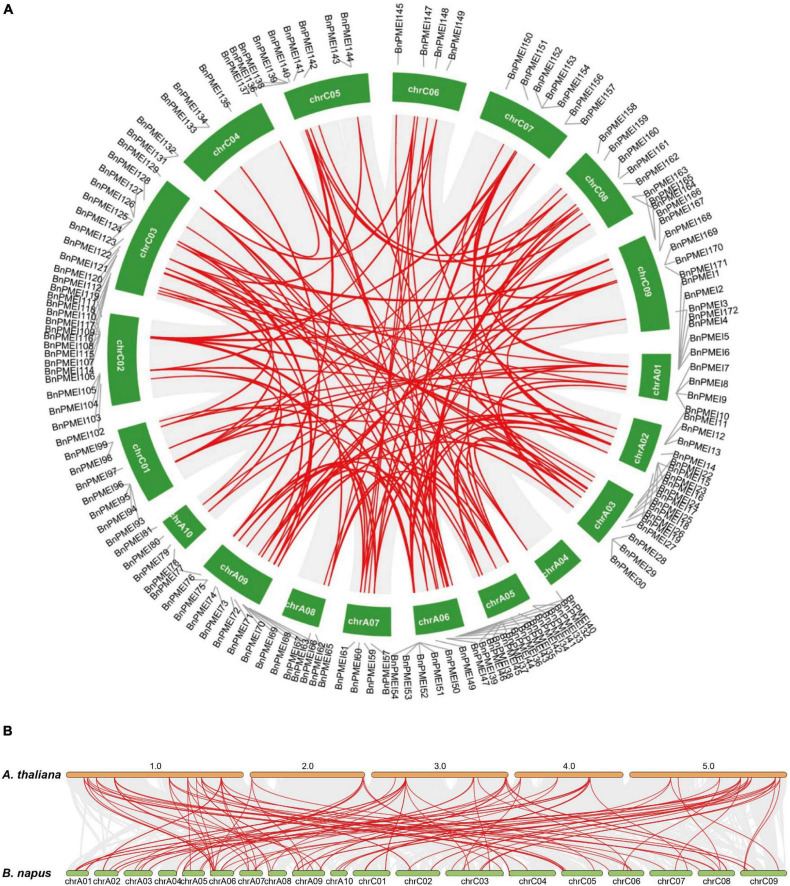
**(A)** Synteny analysis of *PMEI* family in *B. napus*. Gray lines indicated all synteny blocks in the *B. napus* genome, and red lines highlighted the duplicated *BnPMEI* gene pairs. ID of chromosome was indicated in the middle of each chromosome. **(B)** Synteny analysis of *PMEI* family between *B. napus* and *A. thaliana*. Gray lines in the background indicated the collinear blocks within *B. napus* and *A. thaliana* genomes, while red lines highlighted the syntenic *PMEI* gene pairs. The specie names with the prefixes ‘*A. thaliana*’ and ‘*B. napus*’ indicated *Arabidopsis thaliana* and *Brassica napus*, respectively. ID of the *A. thaliana* chromosome was indicated on the top of each chromosome, and ID of *B. napus* chromosome was indicated at the bottom of each chromosome.

Gene duplication is likely essential for adaptive evolution and plays a significant role in the expansion of gene families (Panchy et al., 2016). In this study, duplication events occurred in the *B. napus PMEI* gene family were investigated. As shown in Supplementary Table 4, 123 *BnPMEI* genes were derived from whole-genome duplication (WGD) or segmental duplications. 49 *BnPMEI* genes evolved under dispersed duplication events which may involve repetitive sequences and/or replicative transposition by transposable elements (TEs). Only 9 *BnPMEIs* appeared as a result of tandem duplication. Using MCScanX methods, 166 paralogous gene pairs were identified. Among them, 165 gene pairs were detected across different chromosomes, while only one duplication event occurred within the same chromosome (BnaC04g00440D/BnaC04g51500D) (Figure 4A and Supplementary Table 5a). Additionally, 33 duplication events took place on the AA subgenome, 25 events occurred on the CC subgenome, and 108 across AA/CC subgenomes (Figure 4A and Supplementary Table 5a). The results suggest gene duplications, mainly driven by WGD or segmental events, play a key role in the expansion of *PMEI* gene family in *B. napus*. To characterize the selective pressure on the duplicated *BnPMEI* genes during the evolutionary process, *K*a/*K*s ratios were calculated for the paralogous gene pairs in *B. napus*. Except for one duplication event with a *K*a/*K*s ratio over one, the ratios of *K*a/*K*s for other duplication events were less than one, implying the main driving force for *BnPMEI* family evolution was the negative selection (Supplementary Table 5a). Estimation of divergence-time (Million Years Ago, MYA) revealed the divergence of *PMEIs* in *B. napus* occurred during ∼61.44 MYA (Supplementary Table 5a).

Furthermore, synteny analysis of the *PMEI* gene families between *B. napus* genome and *A. thaliana* genome was performed. Collinearity analysis revealed 105 *BnPMEIs* exhibited syntenic relationships with *AtPMEIs*, of which some *BnPMEIs* were related with more than one orthologous copy in *A. thaliana*, such as *BnPMEI2*, *BnPMEI3*, and *BnPMEI33* etc. (Figure 4B and Supplementary Table 5b). The majority of the orthologous gene pairs between *B. napus* and *A. thaliana.* had a *K*a/*K*s ratio of less than 0.5, implying the *PMEI* gene family might have undergone robust purifying selective pressure during evolution. Analysis of the divergence time of homologous gene pairs between *B. napus–A. thaliana* suggested the divergence of *PMEIs* occurred during 8.77 ∼ 69.3 MYA (Supplementary Table 5b).

### *Cis*-elements in the promoters of *BnPMEI* genes

*Cis*-acting elements play an important role in regulating gene expression. In order to understand the potential regulatory mechanisms of *BnPMEI* genes, cis-elements within the 1.5-kb upstream from ATG for each of the *BnPMEI* gene were analyzed using PlantCARE. A wide range of cis-acting elements were identified in the promoter regions of *BnPMEI* genes, including elements related to plant growth and development, abiotic and biotic stress responses, hormones responses, and basic promoter elements in eukaryotes such as CAAT-box and TATA-box (Supplementary Figure 3 and Supplementary Table 6). Plant hormone-responsive elements were detected in the promoters of a large number of *BnPMEI* genes, including GARE-motif and P-box (gibberellin-responsive elements), CGTCA-motif/TGACG-motif (MeJA-responsiveness), AuxRR-core (auxin responsiveness), ABRE motif (abscisic acid responsiveness), TCA-element (salicylic acid responsiveness), TGA-element (auxin-responsive element) and TATC-box (gibberellin-responsiveness). The most abundant ABRE-motif appeared in the promoter region of 147 *BnPMEI* genes, followed by the MeJA-responsive motifs which were detected in 124 members. In contrast, auxin-related element AuxRR-core appeared in only 25 *PMEI* genes in *B. napus*, and gibberellin-responsive TATC box presented in 29 members. Another important category of cis-elements were environmental stress-responsive elements. ARE motif, essential for the anaerobic induction, appeared in 164 members from the *PMEI* family. LTR, responsive to low temperate, was occupied by 80 *BnPMEI* genes. *Cis*-element essential for drought induction was detected in the promoter region of up to 78 *BnPMEI* genes. A total of 75 *BnPMEI* genes owned TC-rich repeats which are involved in defense and stress response. 62 *BnPMEI* genes carried MYB binding site (MRE) which is involved in light responsiveness. However, the numbers of *BnPMEI* genes containing cis-elements associated with elicitor-mediated activation, anoxic specific induction and wound responsiveness were 14, 8, and 8, respectively (Supplementary Figure 3 and Supplementary Table 6).

A diversity of *cis*-elements related to plant growth and development were detected. For example, light-responsive elements were found in 157 members from the *PMEI* gene family. Another development related *cis*-element was CAAAGATATC motif which is involved in circadian control. *Cis*-elements associated with endosperm expression and meristems expression were detected, implying genes containing these elements were likely to regulate cell wall pectin methylesterase/de-methylesterase during seed germination and cell division. Elements participating in flavonoid biosynthetic gene regulation, differentiation of the palisade mesophyll cells, zein metabolism regulation, seed specific regulation and cell cycle regulation were also detected (Supplementary Figure 3 and Supplementary Table 6).

### Evaluation of disease development in the R-line and S-line

**FIGURE 5:**
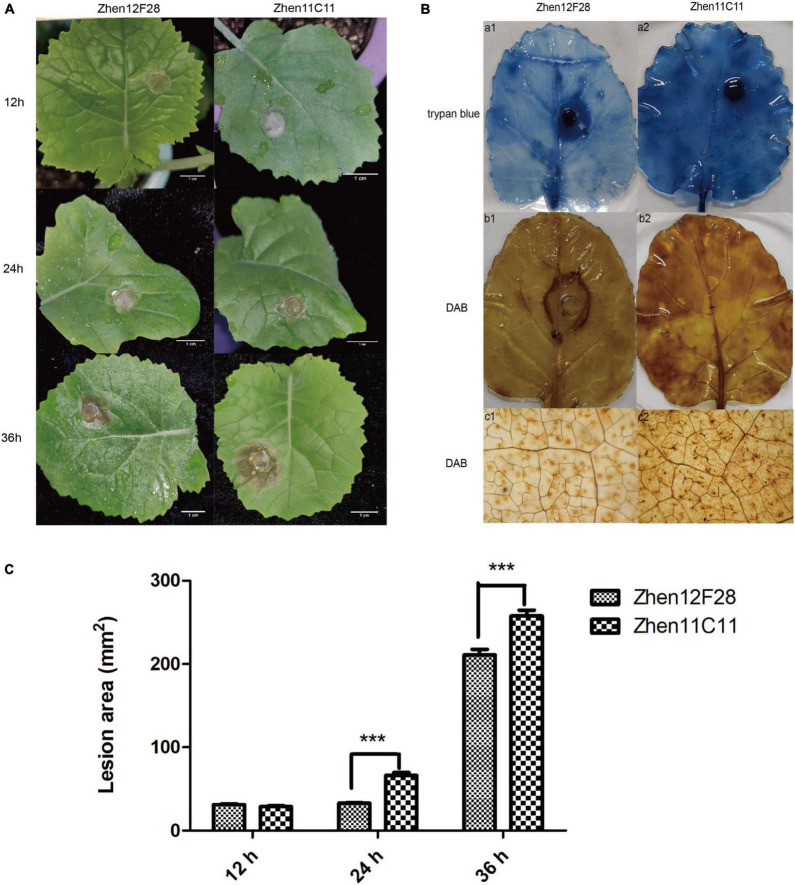
Evaluation of disease development. **(A)** Disease symptoms of leaves in Zhen12F28 (R-line) and Zhen11C11 (S-line) 12, 24, and 36 h post inoculation with *S. sclerotiorum*. Scale bar = 1 cm. **(B)** Trypan blue and DAB staining in the leaves of Zhen12F28 (R-line) and Zhen11C11 (S-line) at 36 h after inoculation. a1 and a2: Trypan blue staining; b1 and b2: DAB staining; c1 and c2: DAB staining imaged under microscope. **(C)** Lesion area of inoculated leaves in Zhen12F28 and Zhen11C11 at 12, 24, and 36 h post inoculation. Error bars are mean ± SEM, *n* = 3. Significant differences between the two lines at each time point are denoted as follows: ***P < 0.001.

Disease symptoms were recorded at each time point after inoculation. For both R-line and S-line, the soft-rotting necrosis occurred as early as 12 h after inoculation with no significant difference detected for the lesion area. Disease symptoms developed rapidly as infection proceeded, and lesion size became apparent at 24 h in both lines (Figure 5A and Supplementary Table 7). Lesion area was significantly larger in the S-line than the R-line at both 24 h and 36 h post inoculation (Figure 5A and Supplementary Table 7). In order to further compare the cellular changes in both lines, trypan blue and DAB staining was performed. Trypan blue staining was conducted to examine cell viability, which was based on the principle that viable cells possessing intact cell membranes could exclude the dyes while died cells could not (Strober, 2015). Inoculated leaves from the S-line showed darker blue color than the R-line, suggesting more died cells were present in the S-line (Figure 5B). The presence and distribution of H_2_O_2_ in leaf cells were detected by DAB staining. DAB can be oxidized by H_2_O_2_ in the presence of some haem-containing proteins, such as peroxidases, to generate a dark brown precipitate which can be visualized as a stain using a microscope. Micrographs of the DAB-stained leaves from the S-line showed denser dark brown precipitates compared to the R-line, indicating higher level of H_2_O_2_ was accumulated during the infection process in the S-line (Figure 5B). The DAB staining result might reflect a more robust defense response associated with reactive oxygen species (ROS) in the S-line compared to the R-line. Lesion area was measured using Image J, and a significant difference (*P* < 0.001) was observed at 24 and 36 h post inoculation, respectively, between the R-line and S-line (Figure 5C and Supplementary Table 7).

### Expression profiles of *BnPMEI* genes in response to *Sclerotinia sclerotiorum* infection

In order to investigate if *BnPMEI* genes were associated with resistance to *S. sclerotiorum*, expression patterns of the *PMEI* gene family were evaluated in our study. Firstly, public transcriptome data was used to evaluate the overall expression patterns of *BnPMEI* family. Transcriptome data was obtained from the NCBI SRA database under the following projects (ID PRJNA274853; PRJNA321917) (Supplementary Table 8a: PRJNA274853; Supplementary Table 8b: PRJNA321917). Wu et al. (2016) reported a global transcriptomic analysis of two *B. napus* pure lines J964 (resistant line, designated the R-line) and J902 (susceptible line, designated the S-line) at 24, 48, and 96 h post-inoculation by the *S. sclerotiorum* isolate on the primary stem; The relative differentially expressed *BnPMEI* genes between resistant and susceptible lines were characterized. In detail, transcripts of three genes including *BnPMEI161*, *BnPMEI64*, and *BnPMEI141* were strongly enhanced at 96 h post inoculation in the S line (Supplementary Figure 4 and Supplementary Table 8a). The three genes were also upregulated in the R-line although to a lesser extent than the S-line at the same timepoint, while they were induced to a higher level at 48 h than 96 h in the R line (Supplementary Figure 4 and Supplementary Table 8a). Expression of *BnPMEIs* were also profiled using RNA-seq data generated from leaf samples treated by *S. sclerotiorum* for 24 h in susceptible (cv. Westar) and tolerant (cv. Zhongyou 821) lines (Girard et al., 2017). A number of *PMEI* genes were up-regulated in both lines, including *BnPMEI33*, *BnPMEI41*, *BnPMEI165*, *BnPMEI164*, *BnPMEI131*, *BnPMEI167*, *BnPMEI76*, and *BnPMEI145* (Supplementary Figure 4 and Supplementary Table 8b).

### Expression levels of 10 *BnPMEI* genes at various infection stages in R line and S line

**FIGURE 6:**
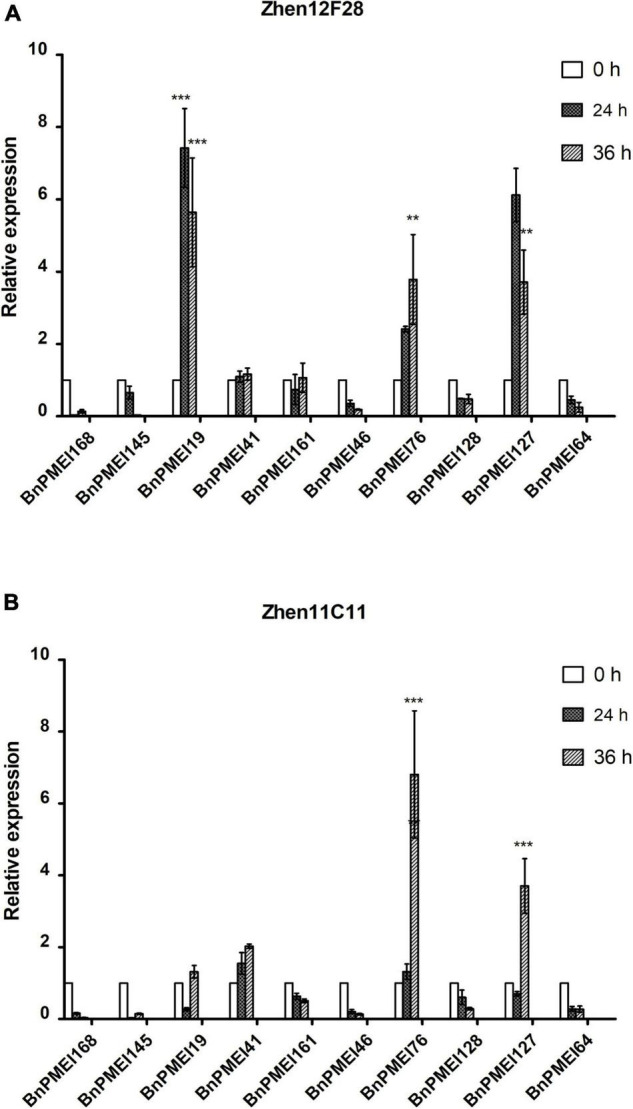
Expression profiles of selected ten *BnPMEI* genes in Zhen12F28 (R-line) **(A)** and Zhen11C11 (S-line) **(B)** after *S. sclerotiorum* infection. The relative expression levels were analyzed by qRT-PCR. Error bars are mean ± SEM, *n* = 3. Significant differences between infected lines and the control (WT) line (0 h) at each time point are denoted as follows: ***P < 0.001; **P < 0.01.

Based on the results of RNA-seq analysis, a total of ten *BnPMEI* genes were selected for further validation. These genes selected for qRT-PCR analysis include *BnPMEI168, BnPMEI145, BnPMEI19, BnPMEI41, BnPMEI161, BnPMEI46, BnPMEI76, BnPMEI128, BnPMEI127*, and *BnPMEI64* (Supplementary Table 2). We used qRT-PCR to examine their expression levels at various infection stages in our R-line and S-line. In general, several *BnPMEI* genes were responsive to *S. sclerotiorum* infection in both lines. In the R-line, *BnPMEI19* was most significantly (*P* < 0.001) induced by *S. sclerotiorum* at both 24 and 36 h after inoculation, followed by *BnPMEI127* and *BnPMEI76* (Figure 6A). In the S-line, *BnPMEI76* showed the largest fold change relative to control (0 h) at 36 h (*P* < 0.001), and transcripts of *BnPMEI127* was four times the amount of control (*P* < 0.001) (Figure 6B). Another gene *BnPMEI41* was also up-regulated in both lines, although to a lesser extent than *BnPMEI19, BnPMEI127*, and *BnPMEI76*. The common targets observed in both lines are likely to be vital for enlightening tolerance to SSR probably *via* strengthening cell wall mechanics and maintaining cell wall integrity (CWI). In contrast, some *BnPMEI* genes were down-regulated during infection, suggesting they might play a negative role in maintaining CWI probably through inducing cell wall loosening.

### Tissue-specific expression patterns of *BnPMEI* genes

Expression profiles of *BnPMEIs* in different tissues was analyzed. The transcript levels of 190 *BnPMEI* genes in five different tissues including root, stem, leaves, petals and silique pericarp at full-bloom stage from *B. napus* cultivar ZhongShuang 11(ZS11) were obtained from public resource Brassica EDB (see footnote 16). A heatmap was constructed to illustrate the global expression patterns of the *BnPMEI* gene family (Supplementary Figure 5). A total of 50 *BnPMEI* genes were specifically expressed in petal with high levels while exhibited low expression levels across other tissues. Six *BnPMEI* genes were merely highly expressed in root, including *BnPMEI87*, *BnPMEI56, BnPMEI8, BnPMEI146, BnPMEI13*, and *BnPMEI123.* Genes that were expressed only in stem including *BnPMEI15, BnPMEI62, BnPMEI49, BnPMEI129, BnPMEI43*, and *BnPMEI175*. The numbers of *BnPMEI* genes that were detected in leaf and silique were 10 and 19, respectively. Numerous genes were expressed in more than one tissue with varying levels of transcripts. The variety of expression patterns suggested a broad range of biological functions of the *BnPMEI* genes during the development of *B. napus.*

**FIGURE 7:**
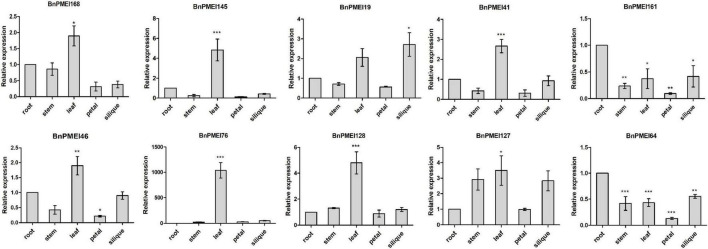
Expression profiles of the selected ten *BnPMEIs* in five different tissues including root, stem, leaf, petal, and silique at full-bloom stage of *B. napus*. The relative expression levels were analyzed by qRT-PCR and expressed as a ratio relative to that of root. Error bars are mean ± SEM, *n* = 3. Significant differences between different tissues and root are denoted as follows: ***P < 0.001; **P < 0.01; *P < 0.05.

qRT-PCR was performed to examine the expression levels of the selected *BnPMEI* genes, in various organs including root, stem, leaf, petal and silique at full-bloom stage of *B. napus* ZS11 cultivar. Significant differences were detected between different tissues for each of the ten genes. As displayed in Figure 7, seven out of ten *BnPMEI* genes showed the highest expression level in leaf. Two BnPMEI genes including *BnPMEI161* and *BnPMEI64* had higher levels of transcripts in root compared to other tissues, and *BnPMEI19* maintained the highest expression level in silique. Three genes including *BnPMEI19, BnPMEI76*, and *BnPMEI 127* were significantly induced in leaves by *S. sclerotiorum* infection, and they also maintained relatively high constitutive expression levels in leaf tissue. This suggests a role of the three *BnPMEI* genes in regulating both plant development and biotic stress. Interestingly, *BnPMEI76* was only detectable in leaf tissue, which was consistent with the public RNA-seq data. Other genes were detected in multiple tissues, suggesting they might function in various tissues during development.

### Chemical analysis of pectin and its degree of methylesterification

**FIGURE 8:**
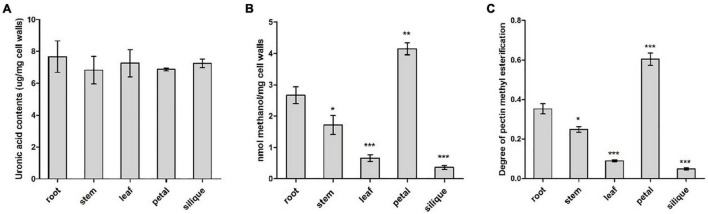
Biochemical analysis of pectin modification in various tissues including root, stem, leaf, petal and silique at full-bloom stage of *B. napus*. **(A)** Analysis of uronic acid content in pectin-enriched fractions of cell walls isolated from different tissues. **(B)** Quantification of cell wall-bound methanol in different tissues. **(C)** Degree of pectin methylesterification of cell walls in different tissues. Error bars are mean ± SEM, *n* = 3. Significant differences between different tissues and root were denoted as follows: ***P < 0.001; **P < 0.01; *P < 0.05.

Pectin content and degree of pectin methylesterification were compared between five different tissues from *B. napus*, including root, stem, leaf, petal and silique at full-bloom stage. Cell wall materials (AIS) was prepared from various tissues and PEFs were isolated from AISs. Pectin content was determined indirectly by measuring uronic acid content in the PEFs as uronic acid is the basic composition of pectin. Similar levels of uronic acid (*P* > 0.05) were detected in all of the five tissues, ranging from 6 to 8 μg/mg cell walls (Figure 8A). It is likely that the level of methyl groups bound to HG and the degree of pectin methylesterification reflects the endogenous PME/PMEI activity. As an indirect reflection of PME/PMEI activities, cell wall-bound methyl ester contents were compared between root and other tissues, respectively, by measuring methanol that was released from cell walls after chemical treatment. Resulted indicated the highest level of methanol was present in pectin fractions isolated from petal cell walls, followed by root and stem. In contrast, PEFs from silique cell walls were observed to have the lowest level of methanol (Figure 8B). This suggested degree of pectin methylesterification was higher in vegetative tissues and flower than fruit tissue. Root tissue was randomly selected as the control group for pairwise comparison. Results revealed significant differences (*P* < 0.05) between root and each of the other four tissues, in terms of both cell wall-bound methanol and degree of HG methylesterification (Figures 8B,C). The results suggested pectin methylesterification status, a major *in muro* modification of pectin, acted as a key determinant of organ and tissue development probably through regulating cell wall mechanics and integrity.

### Gene ontology analysis and proten–protein interactions prediction

GO terms for each BnPMEI protein were determined using Interpro Scan 5. Each BnPMEI protein was annotated with the Molecular Function term GO:0004857, which was defined as “enzyme inhibitor activity.” Part of the BnPMEI proteins were also annotated with the Biological Process term GO:0043086, a GO term defined as “negative regulation of catalytic activity” (Supplementary Table 9a). None of the BnPMEI proteins were associated to any Cellular Component GO term. The results are consistent with BnPMEI having a function related to the inhibition of the activity of PMEs.

**FIGURE 9:**
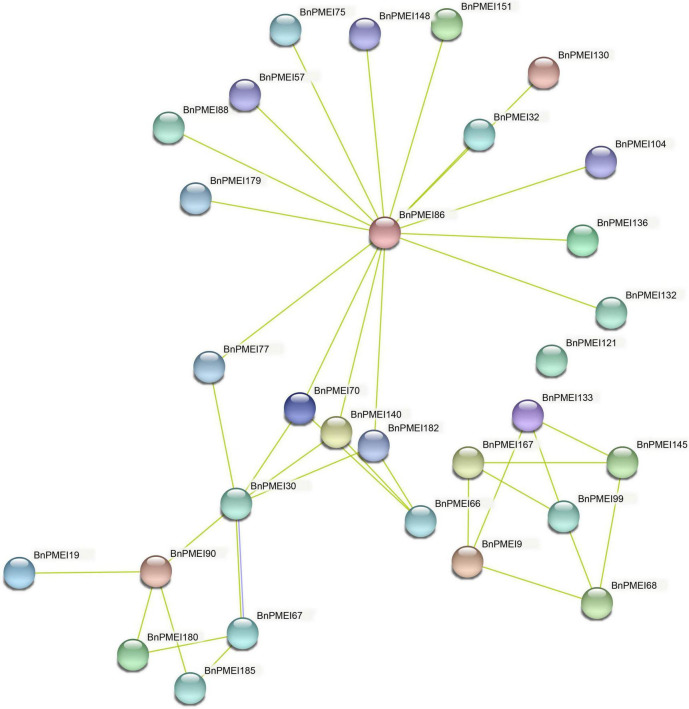
Protein–protein interaction networks of BnPMEIs. The networks were generated from the STRING database. Network nodes represented BnPMEI proteins. Edges represented protein-protein associations.

The STRING database was used to predict potential proteins interacting with each of the BnPMEI protein as well as interaction networks between members of the *BnPMEI* gene family. A total of 30 BnPMEI proteins were involved in PPI networks and interacted with each other (Figure 9 and Supplementary Table 9b). BnPMEI86 had the highest node degree (16), meaning it interacted with 16 BnPMEI proteins, followed by BnPMEI30 with a node degree of 6. In contrast, the majority of the BnPMEIs interacted with one or three BnPMEI proteins. To be specific, 13 BnPMEIs interacted with only one protein and 11 BnPMEIs interacted with three proteins from the *PMEI* family. In addition, each BnPMEI member was checked individually for their interaction networks. Results revealed 47 BnPMEI proteins showed interactions with various proteins in *B. napus*. Detailed information was summarized in Supplementary Table 9c. Numbers of BnPMEIs interacting with 10 proteins and 9 proteins were 18 and 8, respectively. However, 9 BnPMEIs had interaction with only one protein (Supplementary Table 9c). Interestingly, some of these BnPMEIs were detected to have associations with other cell wall structure-related enzymes, suggesting BnPMEI proteins might regulate cell wall metabolism and wall-associated biological processes through their interactions with other cell wall related enzymes. For example, BnPMEI31, BnPMEI87, BnPMEI121 were found to interact with multiple members in the *pectate lyase (PL)* gene family. BnPMEI134 alone was predicted to associate with three types of cell wall related enzymes including cellulose synthase, pectinesterase and xyloglucan endotransglucosylase/hydrolase. Other cell wall related enzymes such as alpha-galactosidase, hexosyltransferase and beta-glucosidase were also among the identifiers involved in these PPI networks (Supplementary Table 9c). In addition to these enzymes related to cell wall metabolism, other genes might be co-expressed with *BnPMEIs* and encode proteins which are involved in cell wall metabolism in a coordinated way with BnPMEIs. These proteins included erecta leucine-rich-repeat receptor-like kinase, thioredoxin, cyclin, thioredoxin, CASP-like protein, bidirectional sugar transporter SWEET, 3-ketoacyl-CoA synthase, peroxidase and proteins in plant LTP family and MIP/aquaporin family (Supplementary Table 9c).

## Discussion

Recent progress on the role of *PMEI* genes has offered new insights into our knowledge of how the degree of HGmethylesterification impacts cell wall properties, plant development, and interactions with their abiotic and biotic environments (Wormit and Usadel, 2018). In this study we have undertaken a comprehensive genome wide analysis of *PMEI* gene family in *B. napus* and have identified 190 PMEIs. This number was apparently higher than previously reported numbers of PMEIs in other dicot species including *A. thaliana* (71) (Wang et al., 2013), *Brassica campestris* (100) (Liu et al., 2018a), *Brassica rapa* (97) (Tan et al., 2018) and flax (95) (Pinzón-Latorre and Deyholos, 2013), as well as monocots such as rice (49) (Nguyen et al., 2016), *Sorghum bicolor* (37) (Ren et al., 2019), *Brachypodium distachyon* (38) (Wolf et al., 2009). *B. napus* contains more PMEI isoforms than other dicots, which might be attributed to the larger size of *B. napus* genome in comparison with other members of the Brassica family. Moreover, in monocots PMEI families are generally of smaller size with respect to number of gene members, likely due to the differences in the cell wall composition. Pectins are generally less abundant and less methylesterified in monocot species in comparison to dicot species (Mohnen, 2008).

The BnPMEIs that were identified were classified into Clades I–V, based on phylogenetic analysis. Intragroup BnPMEIs presented similar patterns of gene structure and motif composition, indicating members from the same clade might originate from a common ancestor and exhibit similar functions. Moreover, this could be a further validation on the phylogenetic classification. Having only one exon was the common pattern within *BnPMEI* genes, which was similar to *A. thaliana* (Wang et al., 2013). Gene duplication is a fundamental process in the evolution of species especially in eukaryotes and plays an important role for the creation of novel gene functions (Lynch and Conery, 2000; Tremblay Savard et al., 2011). Compared with other eukaryotic genomes, plant genomes tend to evolve at higher rates which lead to higher genome diversity (Panchy et al., 2016). Multiple mechanisms contribute to gene duplication. Duplication events are classified as singleton, dispersed, proximal, tandem and whole genome duplication or segmental. Tandem duplication, which takes place locally, results from unequal crossing-over events and leads to a cluster of two or more paralogous sequences with no or few intervening gene sequences (Zhang, 2003). In contrast to tandem duplication, other subgenomic duplication mechanisms result in dispersed duplicates. The analysis of synteny in this study showed whole genome duplication or segmental duplication was the predominant mechanism accounting for the *BnPMEI* gene family expansion. This was in line with the recognized conclusion that the major cause of expansion of gene families in many angiosperms were WGD events (Tang et al., 2008). Calculation of *K*a/*K*s values between paralogous and orthologous pairs indicated that *PMEIs* in *B. napus* and *A. thaliana* were mostly under stabilizing selection except a few sites have undergone positive selection. The only one paralogous gene pair in *BnPMEI* family with a *K*a/*K*s value higher than one could be interpreted as a consequence of very recent duplication, meaning enough time has yet to be elapsed for the related mutations to be silenced.

*Cis*-elements in the promoter region are fundamental in regulating gene expression. Various *cis*-elements were detected by promoter analysis. Elements that were extensively detected include light-responsive elements, hormone-responsive elements, along with those involved in developmental and environmental responses. *Cis*-elements found in *BnPMEI* genes were consistent with previously reported *PMEI* genes in *Brassica campestris, Sorghum bicolor*, and *Brassica oleracea* (Liu et al., 2018a,b; Ren et al., 2019). Multiple *cis*-elements were detected upstream each *BnPMEI* gene, suggesting each member is likely to be regulated by various factors.

Plant cell wall related genes especially those modulating pectin metabolism have been shown to regulate stress responses (Lionetti, 2015). Numerous studies have shown *PMEI* genes were involved in various environmental stresses through maintaining CWI as well as activating pattern triggered immunity (PTI) (Wormit and Usadel, 2018). *B. napus* is constantly threatened by the disease termed SSR which is caused by the fungal pathogen *S. sclerotiorum*. Cultivating disease-resistant rapeseed varieties is the most cost-effective way to prevent and control SSR.

Molecular mechanisms of *B. napus*–*S. sclerotiorum* interactions are complex, which limits the rate of molecular breeding of rapeseed. Recent studies examining the global transcriptional changes during *B. napus–S. sclerotiorum* interactions have revealed alterations in the expression levels of cell wall degradation-related genes (Chittem et al., 2020; Xu et al., 2021). Polygalacturonase-inhibiting proteins (PGIPs), a group of proteins inhibiting the activity of polygalacturonase (PG), could effectively enhance rapeseed immunity against *S. sclerotiorum* infection (Bashi et al., 2013; Wang et al., 2018, 2021). Both PGIPs and PMEIs are involved in regulating pectin degradation. Several studies have revealed a role of PMEIs in regulating plant immunity in *A. thaliana* and wheat (Lionetti et al., 2007; Volpi et al., 2011). A more recent study showed three PMEIs including AtPMEI10, AtPMEI11, and AtPMEI12 increased disease resistance to *Botrytis cinerea* in Arabidopsis through maintaining CWI (Lionetti et al., 2017). Genetic analysis of loci associated with partial resistance to *S. sclerotiorum*, combined with transcriptome analysis suggested a potential role of *BnPMEIs* in regulating SSR resistance in *B. napus* (Zhao and Meng, 2003; Zhao et al., 2007, 2009).

In this study, we firstly analyzed public transcriptome data to examine the global expression profiling of *BnPMEI* genes in response to *S. sclerotiorum* infection in several rapeseed lines. A number of *BnPMEI* members responsive to infection were screened for further validation. We then investigated the expression patterns of ten selected *BnPMEIs* in local rapeseed lines including one partially resistant and one susceptible line. Prior to performing qRT-PCR, disease development, cell viability as well as H_2_O_2_ produced in the inoculated leaves were compared between the two lines. Significant differences in lesion area, death cell percentage and ROS were detected between R- and S- lines especially at later infection stage, suggesting defense responses were precisely regulated and vary between lines. qRT-PCR was used to test expression levels of the ten *BnPMEI* genes in the local lines ‘Zhen12F28’ and ‘Zhen11C11.” Transcripts of three genes including *BnPMEI76, BnPMEI19*, and *BnPMEI127* were significantly up-regulated during the infection process in both R-line and S-line. This was highly consistent with the results of RNA-seq analysis. However, some members were down-regulated by *S. sclerotiorum* treatment. The contrasting effects of the *BnPMEI* genes on SSR disease resistance in our study demonstrated that different PMEI isoforms are likely to modulate cell wall properties and affect the defense outcome using a range of mechanisms. Contrasting effects of genes modulating HG methylesterification degree, were shown in Arabidopsis, in particular, PME genes. AtPME3 and AtPME17 were significantly induced in A. thaliana leaves upon *B. cinerea* infection, but they had contrary impact on resistance against *B. cinerea* (Raiola et al., 2011; Del Corpo et al., 2020). Susceptibility to *B. cinerea* was significantly reduced in *pme3* homozygous mutant plants which showed decreased PME activity and methylated pectins in comparison with WT plants (Raiola et al., 2011). Further investigation indicated reduced susceptibility of pme3 mutant was mainly due to higher DM of pectin that can impair pathogen colonization rather than inducing constitutive and induced defense responses (Raiola et al., 2011). This suggested AtPME3 was a susceptibility factor required for rapid colonization of the host tissue by *B. cinerea* through modification of pectin structures. In contrast, AtPME17 has been shown to greatly trigger PME activity and significantly contributed to resistance against *B. cinerea* in *A. thaliana* (Del Corpo et al., 2020). Molecular and biochemical mechanism analysis suggested AtPME17 contributed to enhanced resistance to *B. cinerea via* activation of pathogen related defense responses, as well as affecting the rheological properties of pectin by facilitating “egg-box” formation (Del Corpo et al., 2020). Similar to *PME* genes, different PMEI isoforms function diversely during plant development and in response to various stresses. For example, tomato *PME1* was highly expressed in expanding green fruit, but not in ripening fruit. Functional characterization in transgenic plants revealed *PME1* played a negative role in regulating fruit softening probably through maintaining CWI. However, PMEIs that were highly ripening-related positively contributed to fruit softening (Liu et al., 2021). This reflects a close link between temporal-spatial expression of PMEI isoforms and their specific role.

*PMEI* gene expression is temporal-spatially regulated during plant-pathogen interactions. Depending on timing and location of the PMEI isoform, it is postulated that different PMEIs might make a range of contributions to disease resistance depending on mechanism and strategies they adopt. The detailed mechanisms of the regulatory role of the potential candidates need further investigation. Expression profiling of *BnPMEI* genes revealed tissue-specific patterns. The *BnPMEIs* genes were classified into seven groups based on their expression patterns. The largest group contained 50 *BnPMEI* genes which showed petal-specific patterns, indicating these genes might be involved in regulating petal development. Of the ten selected *BnPMEI* genes that were examined by qRT-PCR, significant differences were observed between various tissues for each of them.

Pectin content and level of pectin methylesterification were further examined by chemical analysis. Different tissues contained similar levels of pectin content, while degree of pectin methylesterification differed significantly between them. Strikingly, level of methylesterification in pectin fraction was the highest in petal, compared with other tissues. This was consistent with expression patterns of *BnPMEI* genes in petal, with the highest number of *BnPMEI* genes exhibited petal-specific expression. Higher level of PMEI activity is supposed to result in lower level of PME activity, which further leads to higher level of pectin methylesterification. This might account for the highest degree of pectin methylesterification in petal.

Protein–protein interaction and the associated networks are essential to the majority of cellular and biological processes, and activation of most proteins requires their interactions with other proteins (Athanasios et al., 2017). Analyzing the PPI networks allowed us to hypothesize the evolutionary relationships and predict functionally orthologous proteins between species with conserved pathways. Here, PPI network of each BnPMEI and PPI within the BnPMEI family were predicted using the STRING database. Extensive interactions were predicted to occur between different BnPMEIs, or between BnPMEI and other proteins. Interestingly, cell wall structural-related enzymes were identified in the PPI networks, especially those related to pectin metabolism such as pectate lyase, pectinesterase, and beta-glucosidase. This not only suggests pectin metabolism is regulated by the combined action of multiple enzymes, it also supports the recently revised plant cell wall model where pectin plays a much more important role in cell wall mechanics. Previously, the groups of cell wall polysaccharides and the associated enzymes are often discussed as independent entities, but there is strong evidence for close associations among the different classes of molecules (Anderson and Kieber, 2020). Thus, future work on synergistic effect of multiple cell wall-related genes on cell wall integrity and wall associated biological processes is needed. The genome wide analysis of *PMEI* family in *B. napus* provides a theoretical basis for further function characterization and facilitates searching for candidate *PMEI* genes associated with stress response.

## Data availability statement

The original contributions presented in this study are included in the article/Supplementary Material, further inquiries can be directed to the corresponding author.

## Author contributions

DW conceived the original research plans, conducted the bioinformatic analysis, and wrote the manuscript. SJ conducted the experiments, analyzed the data, and involved in writing the manuscript. YS and LL performed the *S. sclerotiorum* inoculation experiments. DW and ZC were involved in reviewing and editing the manuscript. All authors contributed to the article and approved the submitted version.

## Conflict of interest

The authors declare that the research was conducted in the absence of any commercial or financial relationships that could be construed as a potential conflict of interest.

## Publisher’s note

All claims expressed in this article are solely those of the authors and do not necessarily represent those of their affiliated organizations, or those of the publisher, the editors and the reviewers. Any product that may be evaluated in this article, or claim that may be made by its manufacturer, is not guaranteed or endorsed by the publisher.

## Footnotes

1
http://plants.ensembl.org/index.html

2
http://www.genoscope.cns.fr/brassicanapus

3
https://www.arabidopsis.org/

4
https://www.ebi.ac.uk/Tools/hmmer/

5
http://pfam.janelia.org/

6
http://smart.embl-heidelberg.de/

7
https://web.expasy.org/compute_pi/

8
http://www.cbs.dtu.dk/services/SignalP/

9
http://www.cbs.dtu.dk/services/TMHMM/

10
https://wolfpsort.hgc.jp/

11
http://linux1.softberry.com/berry.phtml?topic=protcomppl&group=programs&subgroup=proloc

12
http://meme-suite.org/tools/meme

13
http://gsds.cbi.pku.edu.ch

14
https://itol.embl.de/

15
http://bioinformatics.psb.ugent.be/webtools/plantcare/html/

16
https://brassica.biodb.org/

17
http://www.ebi.ac.uk/interpro/

18
http://gads-gao.lab-org/

## Abbreviations

HG: homogalacturonan
PMEs: pectin methylesterases
PMEIs: pectin methylesterase inhibitors
CWI: cell wall integrity
WGD: whole-genome duplication
RG-I: rhamnogalacturonan I
RG-II: rhamnogalacturonan II
SSR: sclerotinia stem rot
Cd: cadmium
HMM: Hidden Markov Model
MW: molecular weight
pI: isoelectric point
ML: maximum likelihood
DAB: 3,3’-diaminobenzidine
AA: amino acids
TEs: transposable elements
H_2_O_2_: hydrogen peroxide
ROS: reactive oxygen species
GO: Gene Ontology
PL: pectate lyase
ABA: abscisic acid
PTI: pattern triggered immunity
PGIPs: polygalacturonase-inhibiting proteins
PG: polygalacturonase.
