# Supplementary material for: Genome-wide identification of the pectin methylesterase inhibitor genes in Brassica napus and expression analysis of selected members
Source: Front Plant Sci. 2022 Jul 22;13:940284. doi: 10.3389/fpls.2022.940284 (PMC9354821; doi:10.3389/fpls.2022.940284)
Supplement: Supplementary file 1 [file Data_Sheet_1.pdf]

Supplemental materials

Figure S1. Phylogenetic relationships, architecture of conserved motifs and gene structure of BnPMEIs. (a) Phylogenetic tree of 190 BnPMEI proteins (b) The motif composition of BnPMEI proteins. The motifs, numbers 1-5, were displayed in different colored boxes. The sequence information for each motif was provided in Figure S2. The length of protein was estimated using the scale at the bottom (c) Exon/intron structure of *BnPMEI* genes. Yellow boxes represented UTR region and green boxes represented exons. Introns were indicated as black lines. The length of exons/introns can be inferred by the scale at the bottom.

Figure S2. Sequence information of the conserved motifs in BnPMEI proteins.

Figure S3. Cis-elements in the promoters of *BnPMEI* genes.

Figure S4. (a) Expression profiles of *BnaPMEI* genes upon *S. sclerotiorum* infection in two cultivars J902 (Susceptible) and J964 (Resistant) at 3 time points including 24h, 48h and 96h (b) Expression patterns of *BnPMEI* genes in Westar and Zhongyou821 upon *S. sclerotiorum* infection for 24h. Heatmaps were generated based on log 2 of ratios of FPKM values of the treated groups to the control (mocked) groups. The color scale represented relative expression levels from high (red color) to low (blue color). Genes were clustered according to hierarchical clustering.

Figure S5. Expression profiles of *BnaPMEI* genes in various tissues of *B. napus.* Heatmap was generated based on log 2 of FPKM values of five tissues including root, stem, leaf, petal and silique at full-bloom stage.

Table S1. Information of the *PMEI* gene family in *B. napus*.

Table S2. Primers used for qRT-PCR

Table S3. Characteristics of PMEIs identified in the *B. napus* genome.

Table S4. Duplication type of *BnPMEI* genes.

Table S5a. Ka/Ks calculation of the duplicated *PMEI* gene pairs of B. napus; **Table S5b**. Ka/Ks calculation of the duplicated *PMEI* gene pairs of *B. napus* and *A. thaliana*.

Table S6. Cis-elements in the promoter regions of *BnPMEI* genes.

Table S7. Lesion area of inoculated leaves in Zhen12F28 and Zhen11C11 at 12 h, 24 h and 36 h post inoculation.

Table S8a. The expression profiles (ratios of the FPKM values of the treatments to the controls) of *BnaPMEI* genes upon *S. sclerotiorum* infection in susceptible and resistant *B. napus* cultivars; **Table S8b**. The expression profiles (ratios of the FPKM values of the treatments to the controls) of *BnaPMEI* genes in response to *S. sclerotiorum* infection in two *B. napus* cultivars.

Table S9a. GO analysis of each PMEI protein in B. napus; **Table S9b**. PPI network among the BnPMEI proteins; **Table S9c.** The interaction network of each BnPMEI protein.
