# Supplementary figures and images for: Genome-wide identification of the pectin methylesterase inhibitor genes in Brassica napus and expression analysis of selected members
Source: Front Plant Sci. 2022 Jul 22;13:940284. doi: 10.3389/fpls.2022.940284 (PMC9354821; doi:10.3389/fpls.2022.940284)

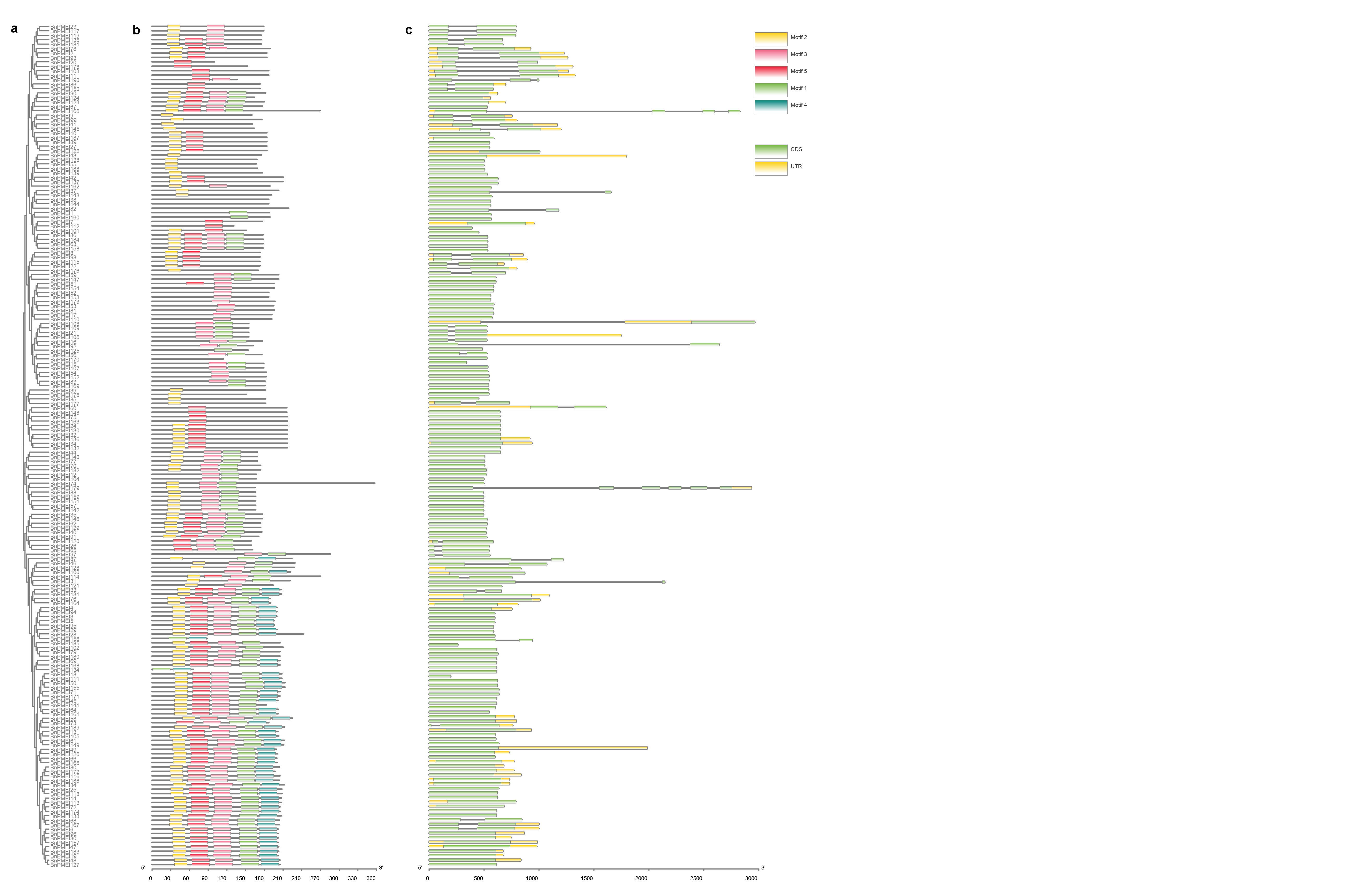

Supplement: Supplementary file 2 [file Image_1.jpg]

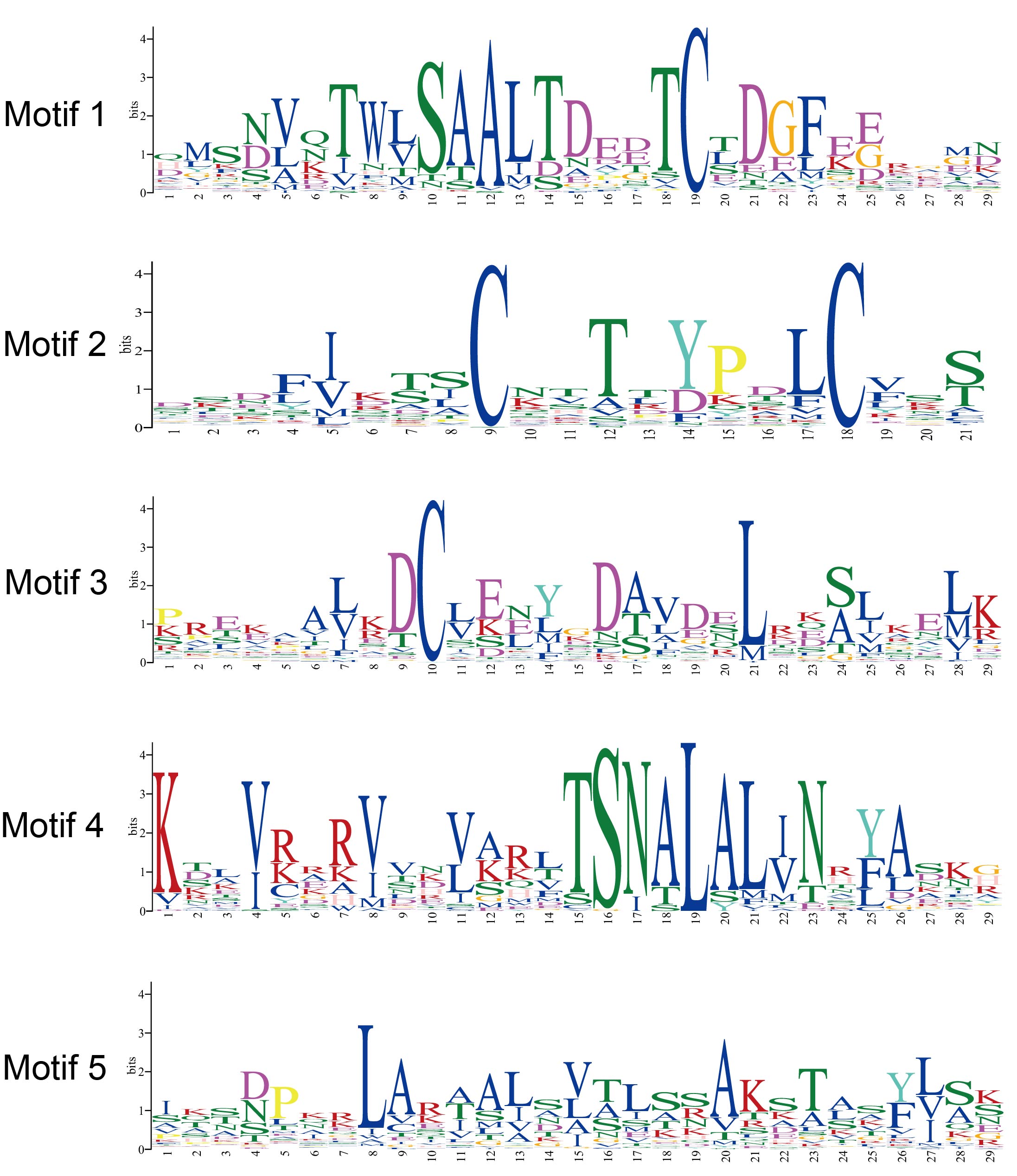

Supplement: Supplementary file 3 [file Image_2.jpg]

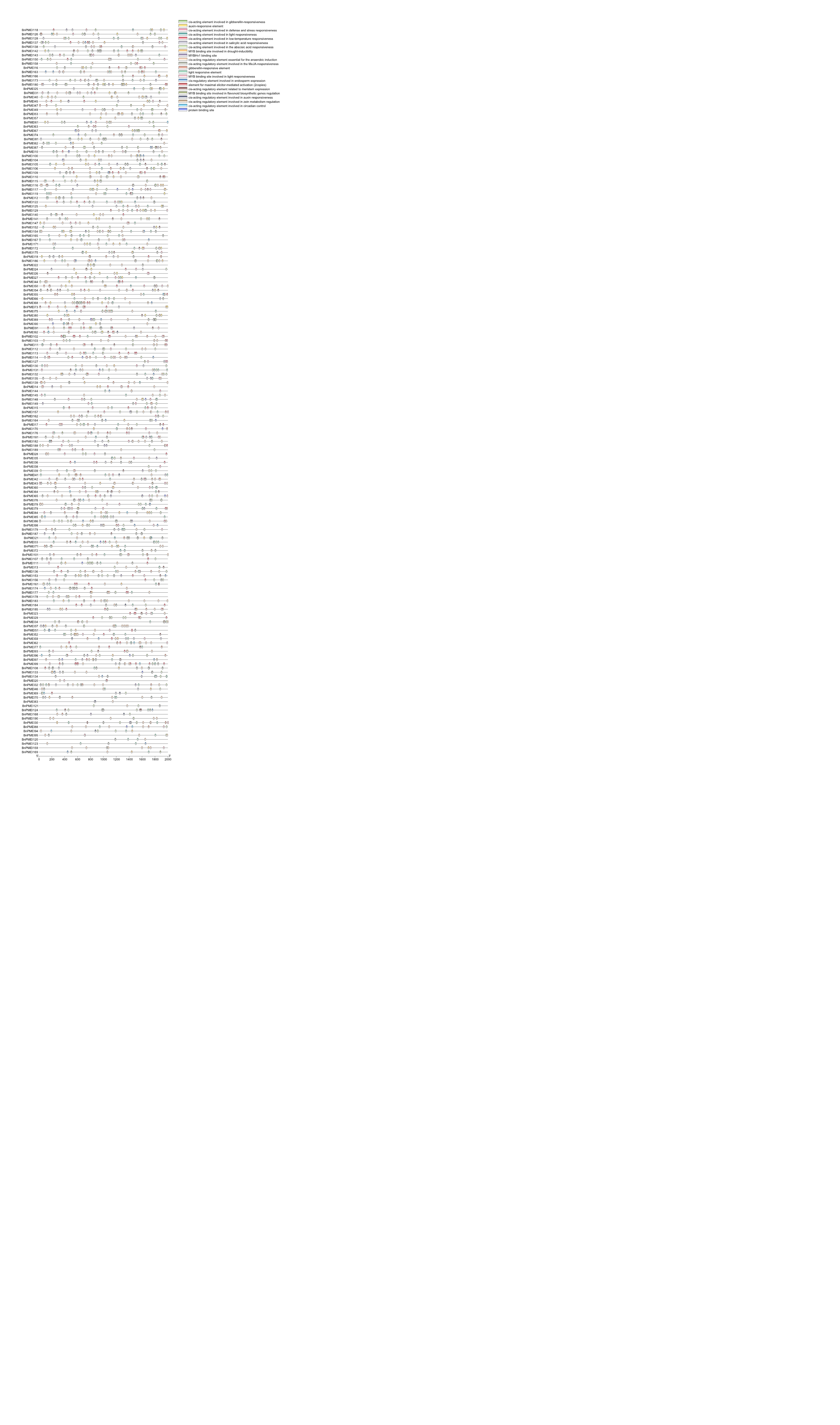

Supplement: Supplementary file 4 [file Image_3.jpeg]

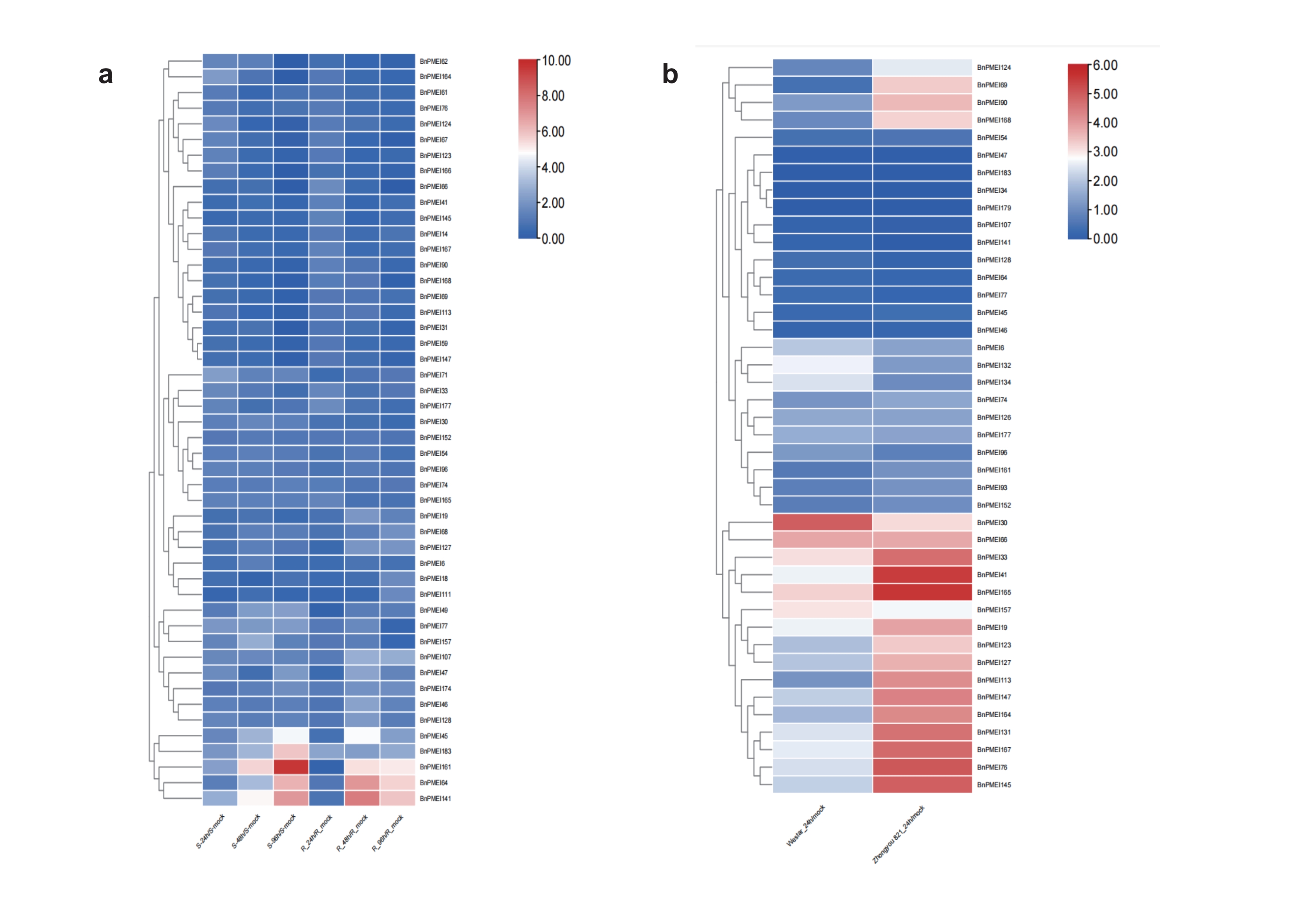

Supplement: Supplementary file 5 [file Image_4.jpeg]

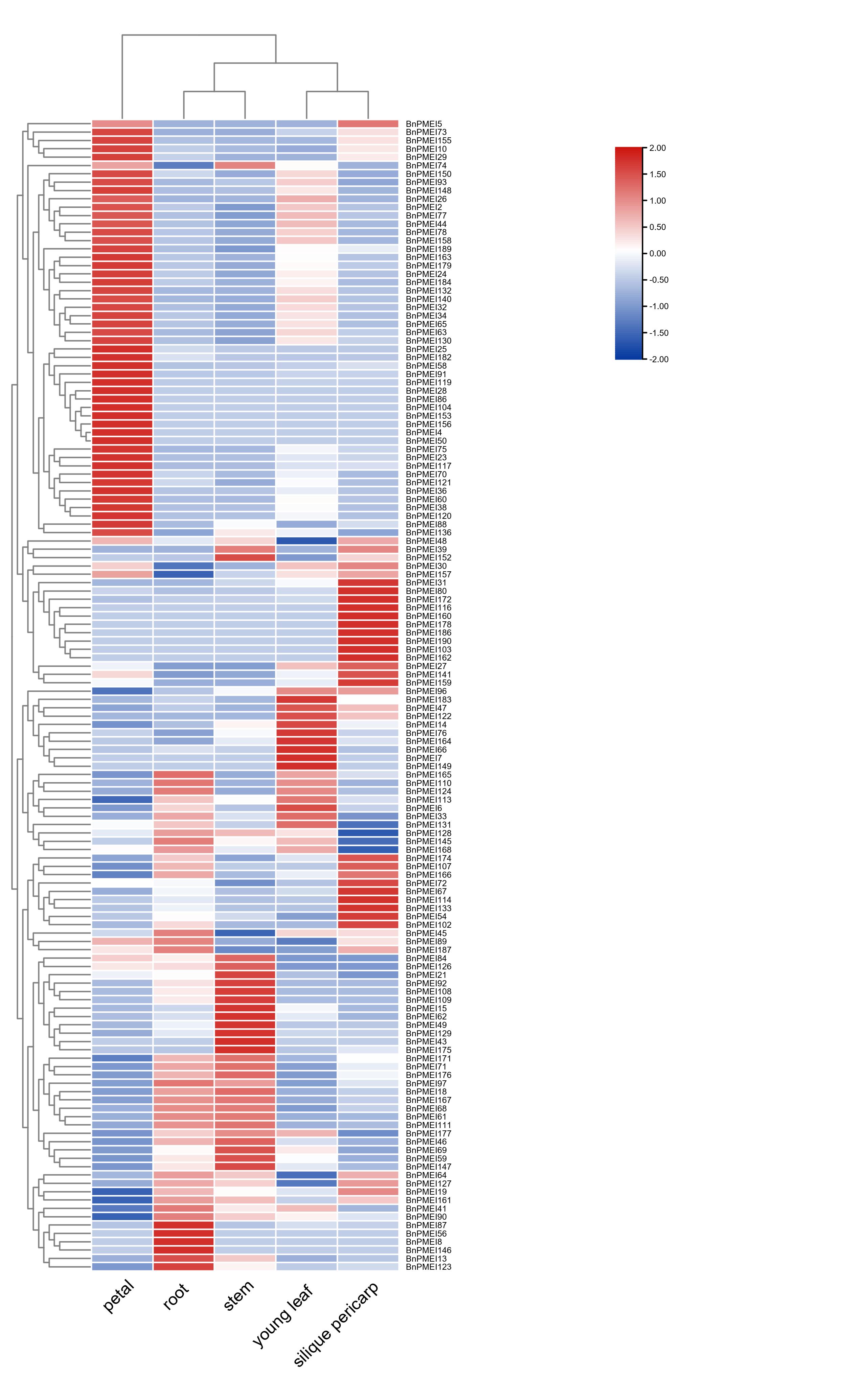

Supplement: Supplementary file 6 [file Image_5.jpg]
