# Supplementary material for: Genome-wide identification of the pectin methylesterase inhibitor genes in Brassica napus and expression analysis of selected members
Source: Front Plant Sci. 2022 Jul 22;13:940284. doi: 10.3389/fpls.2022.940284 (PMC9354821; doi:10.3389/fpls.2022.940284)
Supplement: Supplementary file 8 [file Table_2.docx]

**Table S2 Primers used for qRT-PCR**

| **Gene name** | **Gene ID** | **Primer sequences (5’-3’)** |
| --- | --- | --- |
| BnPMEI168 | BnaC09g13100D | F: CAGCAACTCCATCAACATATTC  R: GGAGATAGAGAGATGTAGCAAAG |
| BnPMEI145 | BnaC06g01490D | F: TGTGGTATGGGTGTTTGATG  R: CAAGAACAGGTAGGCCTTTAG |
| BnPMEI19 | BnaA02g33370D | F: CTATGTGGTAGTGCCAAGAATC  R: CTTGATCCGTCCATCCATAAAC |
| BnPMEI41 | BnaA06g04140D | F: GATAGAGCAGACATGCAAAGAG  R: GTCTAAAGCCTGTTTCAGTTCC |
| BnPMEI161 | BnaC08g16460D | F: GATGAACCACCATCGTCAAG  R: GCTCGTGAATCTCTTAACGTC |
| BnPMEI46 | BnaA06g17230D | F: CTCCAACTCTCTCAAATCAGTATG  R: TGAACGGAAAGACATGGATAAG |
| BnPMEI76 | BnaA09g40170D | F: GAGTTTCAGGAGCAGATGAG  R: TTAGCCGCTAGCTTGTTAAG |
| BnPMEI128 | BnaC03g57120D | F: CAATCCTCGAGCTTTCTCTTC  R: CCTTCTCCGTCATCCAATTC |
| BnPMEI127 | BnaC03g51740D | F: CCATACAAACAAGCCCTAAAC  R: CTCCCACATAGCTTCATCTC |
| BnPMEI64 | BnaA08g31370D | F:GCTTGCTCTAGTCAACACATAC  R: TAGCTTAATGCAACACACAAGC |
| Actin | BnaA03g55890D | F: TGTTGCTATCCAGGCTGTTCTTTC  R: GATAGCGTGAGGAAGAGCATAACC |
