# Supplementary material for: Genome-wide identification of the pectin methylesterase inhibitor genes in Brassica napus and expression analysis of selected members
Source: Front Plant Sci. 2022 Jul 22;13:940284. doi: 10.3389/fpls.2022.940284 (PMC9354821; doi:10.3389/fpls.2022.940284)
Supplement: Supplementary file 13 [file Table_7.docx]

**Table S7 Lesion area of inoculated leaves in Zhen12F28 and Zhen11C11 at 12 h, 24h and 36h post inoculation.** Three independent biological replicates were measured at each time point for both lines.

| **Time point**  **Line** | **12 h** | **24 h** | **36 h** |
| --- | --- | --- | --- |
| Zhen12F28-Line1 | 28.7 | 31.3 | 199 |
| Zhen12F28-Line2 | 31.7 | 33.2 | 210.8 |
| Zhen12F28-Line3 | 32.6 | 33.9 | 222.8 |
| Zhen11C11-line1 | 27.2 | 59.3 | 245.6 |
| Zhen11C11-line2 | 29 | 67.9 | 256.7 |
| Zhen11C11-line3 | 30.6 | 71.2 | 270.4 |
